# A SERS/LSPR Dual-Signal Aptamer Sensor for Abscisic Acid Detection Based on Unmodified Gold Nanoparticles

**DOI:** 10.3390/bios16030152

**Published:** 2026-03-10

**Authors:** Yanyan Zhang, Junjuan Shang, Linze Li, Mengying Du, Hao Zhang, Jiandong Hu

**Affiliations:** 1 College of Mechanical and Electrical Engineering, Henan Agricultural University, Zhengzhou 450002, China; zyanyan0923@henau.edu.cn (Y.Z.); lilinze@henau.edu.cn (L.L.); dmy@stu.henau.edu.cn (M.D.); 2 Henan International Joint Laboratory of Laser Technology in Agricultural Sciences, Zhengzhou 450002, China; 3 State Key Laboratory of Wheat and Maize Crop Science, Zhengzhou 450002, China

**Keywords:** abscisic acid, aptamer sensor, surface-enhanced Raman spectroscopy (SERS), local surface plasmon resonance (LSPR), dual-signals

## Abstract

The plant hormone abscisic acid (ABA) plays an important role in crop growth and development, so it is urgent to establish a simple and sensitive method for the detection of ABA. (1) As one of the most sensitive spectral detection methods, surface-enhanced Raman spectroscopy (SERS) has made some progress in the detection of ABA, but it involved a complicated modification process of noble metal nanoparticles and was time-consuming. (2) In this work, a SERS and (local surface plasmon resonance) LSPR dual-signal aptamer (Apt) sensor based on the aggregation of dispersed (gold nanoparticles) AuNPs and the improved plasmonic coupling with formed SERS was developed and applied to the detection of the plant hormone ABA. Through the specific recognition of Apt and ABA, the prepared crystal violet (CV) and Apt modified AuNPs tended to aggregate in a high concentration salt solution, resulting in changes in LSPR characteristics of the detection system and enhanced SERS intensity of CV signal molecules. Thus, the quantitative relationship between ABA concentration and SERS intensity of signal molecule CV and the degree of absorbance change of AuNPs were established. (3) The linear range detection of SERS was 0.04~40 µM, the detection limit lod (LOD) was 17.6 nM, the linear range detection of LSPR was 0.4~80 µM, and the LOD was 36 nM. (4) The sensor has a good ability to detect ABA in the samples of common plants such as cucumber and tomato and has the characteristics of no chemical bond modification, more reliable detection results, and a universal detection platform.

## 1. Introduction

The plant hormone abscisic acid (ABA) is known to act as an environmental regulator in plants, helping crops cope with droughts, floods, pests, and other harsh conditions [1]. Therefore, ABA detection can help botanists and food experts to grasp the growth status of crops in time and actively respond to and intervene in the disaster of food security brought by harsh environmental factors, which has positive significance for promoting the guarantee of global human food security [2]. At present, the main methods for ABA detection are an enzyme-linked immunosorbent assay (ELISA) and high-performance liquid chromatography (HPLC) [3]. These two methods not only have the advantages of high precision and good specificity but also have the disadvantages of high professionalism and complicated steps [4]. Therefore, the development of emerging, rapid, and low professionalism detection methods become the direction of the ABA detection field.

Surface-enhanced Raman scattering (SERS) is one of the most sensitive spectral methods in the field of bio-sensing detection filed, which is known for its ability to sensitively characterize targets, and has advantages such as a narrow band, fingerprint recognition, and high sensitivity [5,6,7]. Due to its good application at the level of single molecule detection, SERS technology has been widely used in chemical analysis [8], biomedical imaging [9], environmental pollutants, and food detection and other fields [10,11,12]. However, in the field of plant hormone detection application, there are few studies on using SERS technology to detect plant hormones. Regarding the research on the detection of ABA using the SERS method, our research group has conducted some studies and achieved certain results. Firstly, we constructed core-shell nanospheres and gold nanorod structures. Then, modifying the nanostructures with aptamers as the signal probes, we utilized the magnetic nanoparticles which were used as capture probes. Eventually, we constructed SERS aptamer biosensors capable of sensitively detecting ABA [13,14]. However, the modification process involves the construction of a detection system (sandwich competitive structure or magnetic separation method) as well as multi-step centrifugation and washing, which increases the uncertain factors in the modification process [15]. Therefore, it is challenging for direct, simple, and rapid SERS detection, which also limits the wide application of SERS biosensors. Recent studies have found that SERS signal intensity can be controlled and modulated by tuning the degree of plasma coupling between particles. Through tuning, the SERS intensity of probe molecules was significantly enhanced under the action of the electric field, which makes it possible to modulate the ability of electromagnetic fields to enhance intensity by controlling the distance between metal nanoparticles [16,17].

**Scheme 1:**
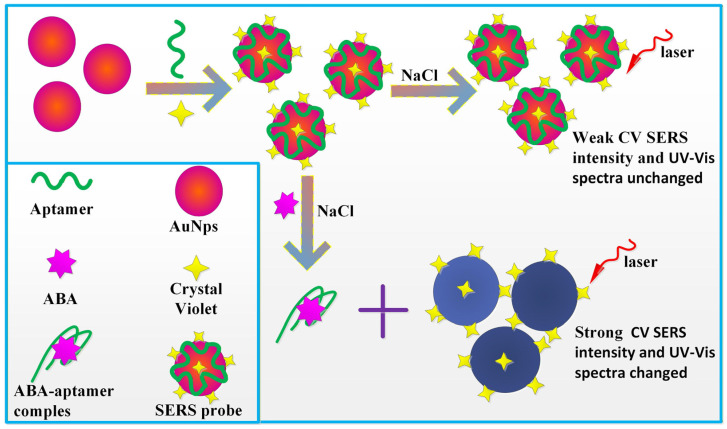
Principle of ABA detection using LSPR/ SERS dual-signal sensor.

In this work, a new LSPR/SERS dual-signal output detection method for ABA was proposed (Scheme 1). First, AuNPs were synthesized using the traditional method.

Then, crystal violet (CV) molecules and Apt of ABA were modified on the AuNPs, CV was attached to the surface of AuNPs by electrostatic attraction, and the Apt was adsorbed on the surface of the AuNPs through strong coordination bonds between base nitrogen and Au atoms. In the absence of ABA in the detection system, because the AuNPs were wrapped by ABA Apt, and after adding high concentration salt solution, the AuNPs could still disperse well under the protection of the Apt, showing bright purplish red. However, in the presence of ABA in the detection system, the specific binding of ABA to the Apt will change the conformation of the Apt and make the Apt free from the AuNPs. The AuNPs, without Apt protection, will aggregate in a high concentration salt solution, the distance between the particles will be decreased, the color of the solution will be changed, and then the LSPR characteristics will change. So the higher the concentration of ABA, the more Apt fell off from the AuNPs, the more exposed AuNPs aggregated in the salt solution, the more changes in solution color and LSPR, and the stronger the SERS signal of CV, indicating that the degree of changes of LSPR and CV SERS intensity were proportional to ABA concentration. The dual-signal aptamer sensor developed using this method showed good sensitivity and anti-interference in ABA detection. Best of all, the sensor does not require additional modification steps, greatly saving time and avoiding the uncertainties in the modification process. Therefore, the proposed dual-signal aptamer sensor can be used as a potential tool for the detection of plant hormones to meet the needs of smart detection.

## 2. Experimental Section

### 2.1. Materials and Instruments

The chlorauric acid (AuCl_3_·HCl·4H_2_O, 99.5%) and trisodium citrate (C_6_H_5_Na_3_O_7_·2H_2_O, 99.0%) used in the synthesis of AuNPs were purchased from Sinopharm Group (Shanghai, China) and were not further purified during use. Other chemicals used in this paper, CV and ABA, were analytically pure, and no further purification was required before use. The ABA aptamer sequence was ATG GGT TAG GTG GAG GTG ATT CCG GGA ATT CGC CCT AAA TAC GAG CAA C oligonucleotide chain composed of 52 bases [18], which was purchased from Sangon Biotech Co., Ltd., (Shanghai, China). The Apt purchased needed to be further quenched before use. For the quenching method used, refer to reference [13]. The commercial ELISA reagent kits were purchased from Shanghai Enzyme-Linked Biotechnology Co., Ltd (Shanghai, China).

The instruments used in this work included a Raman microscopic system (Pioneer Technology Co., Ltd., Beijing, China), a transmission electron microscope (JEM-1400 Plus, JEOL Ltd., Tokyo, Japan) operating at 120 kV, a UV-Vis spectrophotometer (Nanjing Feiler Instrument Co., Ltd., Nanjing, China), with operating wavelengths of 200–1000 nm, and a High speed centrifuge (H1650R, Hunan Xiangyi Centrifuge Co., Ltd., Changsha, China).

### 2.2. Construction of Dual-Signal Sensor

The construction of dual-signal aptamer sensor actually involves modifying the signal molecule CV and aptamer onto AuNPs. Therefore, the construction of the sensor was divided into two steps: (1) The synthesis of AuNPs, and the synthesis method was described in Supplementary Materials “1.1 The method for synthesizing AuNPs” [13]. According to the Lambert–Beer theorem A = εbc, the concentration of AuNPs was calculated. A was the absorbance at the maximum absorption wavelength, ε was the molar extinction coefficient, expressed in M^−1^·cm^−1^, and the unit of optical path length b is centimeter. (2) The aptamer sensor AuNPs@Apt@CV was formed by modifying ABA Apt and signal molecule CV on AuNPs. The Raman characteristic peaks of signal molecule CV were 892, 1165, 1283, 1370, 1422, 1521, 1581, and 1612 cm^−1^, which were, respectively, assigned to radial ring skeleton vibration, C-H bending vibration of ring in plane, C-C stretching vibration in benzene ring, N-benzene ring tensile vibration, C-C stretching vibration and ring deformation of benzene ring, stretching and bending vibrations of benzene rings, C-C stretching vibration and ring deformation of the benzene ring, the stretching vibration between benzene ring and N atom, and the stretching vibration of C-C [19]. Among them, the SERS intensity of the maximum characteristic peak 1612 cm^−1^ was used to quantify ABA concentration in the later quantitative detection test of ABA.

AuNPs@Apt@CV was formed by modifying the Apt and CV on AuNPs. 100 µL 1 µM Apt solution, and 100 µL 0.8 µM CV solution was added in 1 mL AuNPs, incubated at room temperature for 8 min, centrifuged at 8000 rpm for 10 min, and the precipitate was dispersed in 1 mL water to form AuNPs@Apt@CV solution.

### 2.3. Quantitative Detection of Abscisic Acid Using the Dual-Signal Aptamer Sensor

The general steps for detecting ABA using the constructed LSPR/SERS aptamer sensor were as follows: 100 µL of ABA solution of different concentrations (0.04, 0.4, 2, 20, 40, 80 µM) were added to 1 mL AuNPs@Apt@CV solution, incubated at room temperature for 25 min to ensure adequate interaction between aptamer and target molecule ABA. Then, 100 µL, 500 mM NaCl solution was added, and 300 µL pure water was added to the mixture for a total volume of 1.5 mL. After mixing, the UV-Vis spectrum and SERS spectrum of each sample were recorded at room temperature, and each sample was collected three times; the average value was used as the final spectral data, and the spectral curve was drawn.

The experimental steps of real sample detection ability of the sensor were as follows: ABA extracted from cucumber, tomato, wheat, and other common crops was used to replace the standard ABA solution. The methods for obtaining extracts from plant substrates were described in Supplementary Materials “1.2 Methods for obtaining extracts from plant substrates”, and the constructed SERS/LSPR dual-signal aptamer sensor was used for detection, and the detection value was compared with the ELISA kit detection value.

### 2.4. Evaluation of Anti-Interference Capability

Several common hormones in plants, such as gibberellin (GA3), ethylene, vitamin (VC), and β-carotene (β-Car), which may be extracted from plant tissues, were taken as interfering substances. The same experiment was conducted on GA3, VC, β-Car, and ethylene to verify the anti-interference ability of the constructed LSPR/SERS dual-signal aptamer sensor.

## 3. Results

### 3.1. Characterization of Gold Nanoparticles

**Figure 1:**
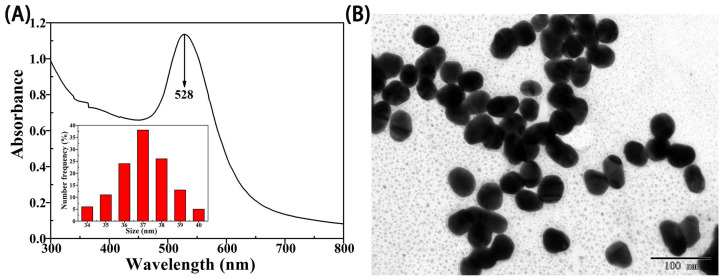
Characterization of gold nanoparticles. (**A**) UV-Vis spectra and (**B**) TEM of AuNPs.

The 37 nm AuNPs (Figure 1A insertion) of the experiment were synthesized, with an SPR peak of 528 nm, which is a narrow peak pattern (Figure 1A). At the same time, the TEM images of AuNPs show that the morphology was uniform and well dispersed (Figure 1B); this is because the surface of AuNPs contains citric acid ions, which generate electrostatic repulsion and ensure the AuNPs have a good dispersion performance. It has been reported that the molar extinction coefficient of 37 nm AuNPs at 528 nm was 2.78 × 108 M^−1^·cm^−1^ [20], and the absorbance at 528 nm was obtained by UV-Vis spectrometer, so the concentration of 7.51 nM of AuNPs was calculated.

### 3.2. Feasibility Verification of Sensor Detection of ABA

The feasibility verification of the constructed dual-signal LSPR/SERS aptamer sensor included the aggregation of well-dispersed AuNPs, conformational changes of Apt before and after binding between aptamers and ABA, and coupling of enhanced plasma formation and SERS.

**Figure 2:**
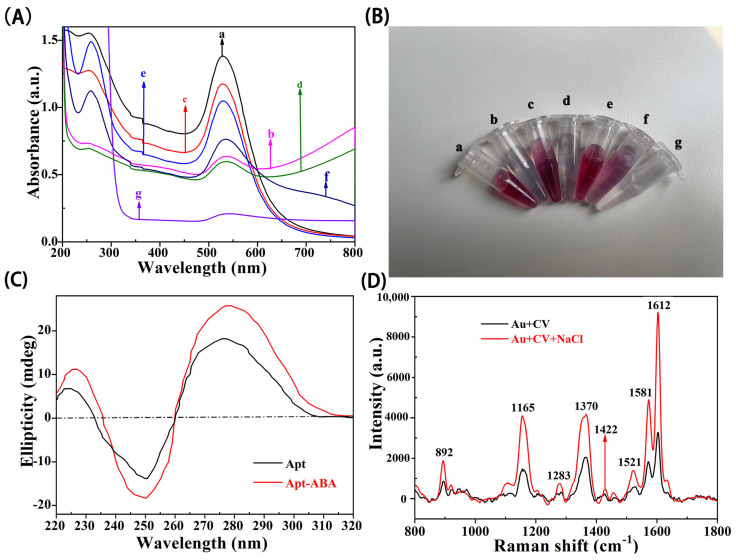
The feasibility verification of the experimental scheme. (**A**) UV-Vis spectra; (**B**) color change of AuNPs of different systems; (a: Au; b: Au + NaCl; c: Au + CV; d: Au + CV + NaCl; e: Au + CV + Apt; f: Au + CV + Apt + NaCl; g: Au + CV + Apt + ABA + NaCl); (**C**) circular dichroism spectra of aptamer before and after binding to ABA; (**D**) SERS spectrum of CV before and after adding sodium chloride.

As shown in Figure 2A,B, on the one hand, well-dispersed AuNPs (Figure 2B a) showed SPR signal peaks at 528 nm (Figure 2A curve a), and once mixed with the salt solution, the electrostatic repulsion between the metal nanoparticles was broken, and the negatively charged AuNPs with citrate root produced a certain degree of aggregation. The absorbance of AuNPs at 528 nm represents the dispersion state of AuNPs. The absorbance decreases significantly with the addition of NaCl solution, and different absorption peaks appear near 620 nm (Figure 2A curve b). Then, we can see in Figure 2B b that the mixture changes rapidly from red to bluish-grey. However, after the introduction of Apt into the system, the absorbance of AuNPs increased to the point of approaching that of mono-dispersed AuNPs (Figure 2A curve e), and the color of the red solution remained unchanged after the addition of the salt solution (Figure 2B e). This phenomenon can be explained by the single stranded free Apt maintaining the irregular helical structure in the mixture, and the nucleic acid group was exposed to the outside, which makes the Apt itself adsorbed on the surface of AuNPs through the strong coordination bond between the base N atom and the gold atom and makes the electrostatic repulsion stronger between the negatively charged phosphate skeleton and the AuNPs [21]. Thus, AuNPs become more stable for salt-induced aggregation and dispersion as the Apt concentration increases and the negative charge increases.

On the contrary, when both Apt and ABA were present in the system solution containing sodium chloride, the absorbance of the solution at 528 nm decreased seriously (Figure 2A curve f), and the mixture appeared blue-gray again (Figure 2B f), interpreted as a specific molecular interaction between the identifying element Apt and the target ABA, resulting in a conformation change in the Apt structure (Apt folding into a G-tetramer structure) [22]. This hypothesis was further confirmed by comparing the variation of Apt circular dichroism (CD) with and without ABA. As shown in Figure 2C, the black curve shows the CD spectrum for Apt alone. The maximum value of positive ovality and minimum value of negative ovality appeared near 279 nm and 251 nm separately, which was due to the aggregation of the nucleic acid bases. After the addition of target ABA, as shown in the red curve, both positive and negative ovality maxima and minima were enhanced due to specific interactions induced by the Apt–ABA complex, which further results in the formation of more G-tetramer structures [23]. Therefore, in this work, the ability of ABA to regulate the variation state of AuNPs aggregation was verified, and the results were consistent with the results of UV-Vis spectroscopy.

On the other hand, different AuNP aggregation states can accelerate the plasma coupling and enhance the Raman spectral electromagnetic field. Compared with the traditional SERS method, CV was used as the Raman signal reporter molecule. The signal transitioned from the typical target ABA peak to CV peak in the SERS spectrum, and CV could clearly reflect the Raman characteristics [24]. Previous studies have shown that Raman labeled molecules between aggregates of noble metal nanoparticles can obtain a greater SERS enhancement factor than dispersed AuNPs [25]. In this paper, CV was selected as a Raman marker for ABA analysis to provide simple and narrow characteristic peaks. CV can be attached to the surface of AuNPs coated with citrate by electrostatic attraction, which means that the constructed biosensor has the advantage of no chemical covalent bonds. When CV was adsorbed on AuNPs and mixed with NaCl, it formed clusters and produced large enhanced Raman signals. As can be seen from Figure 2D, SERS intensity increased after NaCl was added.

### 3.3. The Optimization of the Experimental Parameters

**Figure 3:**
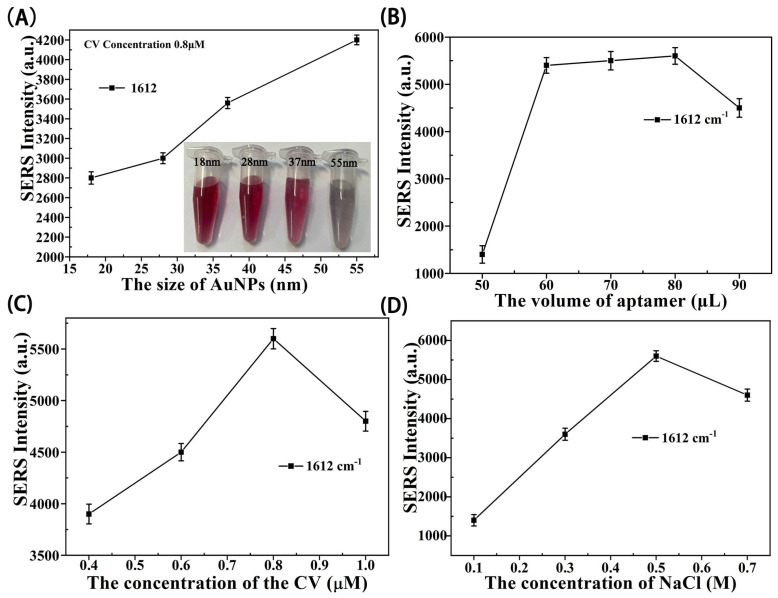
Optimization of (**A**) the AuNPs size and concentrations of (**B**) Apt (**C**) CV and (**D**) NaCl.

The potential parameters influencing the high sensitivity of ABA detection were optimized. Firstly, with the concentration of 1% trisodium citrate increasing from 0.6 mL to 0.8 mL, 1.2 mL and 1.5 mL, the average diameter of the AuNPs decreased from about 55 nm to 37 nm, 28 nm, and 18 nm, respectively. On the one hand, larger nanoparticles can generate stronger enhanced electric fields under laser excitation. But, with the increase in the size of AuNPs, the radiation attenuation becomes more serious [26]. Therefore, the protective ability of Apt and the enhancement ability of CV by using nanoparticles of different sizes in the detection system were compared. As can be seen from Figure 3A, although AuNPs with a diameter of 55 nm showed slightly stronger SERS intensity than AuNPs with a diameter of 37 nm, it was also difficult to protect 55 nm AuNPs from salt-induced aggregation with ABA aptamer after the addition of the salt solution (Figure 3A illustration). That is, more Apt was needed to make larger AuNPs stable in salt water. Therefore, AuNPs with a diameter of 37 nm were selected in this study, which not only had high SERS enhancement but also could well protect AuNPs from salt-induced aggregation due to the presence aptamer of ABA [27].

Secondly, Figure 3B revealed the changes in SERS intensity of the CV signal molecules with the varying concentration of Apt under the same conditions. With the increasing volume of Apt, the SERS intensity first increased and then declined. This was because a small amount of Apt could not sufficiently protect the AuNPs, and the AuNP agglomeration seriously under the effect of salt solution could not effectively enhance SERS. however, more Apt may cause redundancy and compete with ABA molecules in the detection system, thereby hindering the aggregation of AuNPs, and reduceing the SERS intensity of signal CV molecules; thus, an optimized concentration of Apt was obtained as 1 µM, 80 µL.

Thirdly, the concentration of Raman signal molecule CV affected the performance of the sensor. If the concentration of CV was too low, the number of signal molecules adsorbed on the AuNPs was too small, resulting in the SERS signal intensity after the aggregation of AuNPs being very small, and the sensitivity of the Apt sensor constructed would be affected. As can be seen from Figure 3C, with the increase in CV concentration, the SERS signal intensity after the aggregation of AuNPs increased. When the concentration of CV was 1.0 µM, the SERS intensity after the aggregation of AuNPs decreased instead, which may be due to the high CV concentration affecting the encapsulation of Apt with the salt-induced aggregation ability of AuNPs. Therefore, the concentration of CV in this study was determined to be 0.8 µM.

Lastly, the effect of NaCl was investigated in the range from 0.1 M to 0.7 M (Figure 3D). If there was much less salt, dispersed AuNPs could not be efficiently aggregated. Oppositely, excess salt could generate over-loaded background signals. Thus, 0.5 M of NaCl was employed for the following experiment.

**Figure 4:**
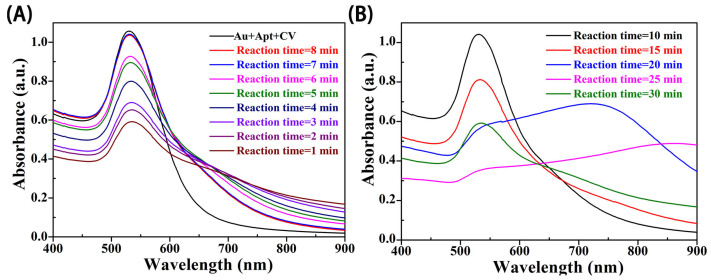
Optimization of the reaction time of (**A**) CV-Apt-AuNPs and (**B**) Apt-ABA.

In addition, the reaction time of Apt-ABA, and CV-Apt-AuNPs were also adjusted to be 25 min (Figure 4B) and 8 min (Figure 4A), ensuring that all the components interacted with each other sufficiently.

### 3.4. The Detection of Abscisic Acid-Based Dual-Signal Aptamer Sensor

**Figure 5:**
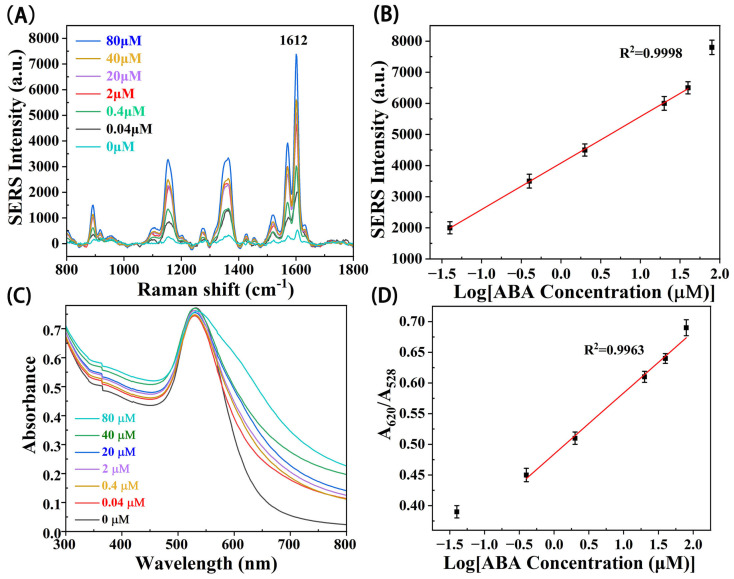
(**A**) SERS spectra and (**C**) UV-Vis Absorption spectra of sensor in the presence of different concentrations (0~80 µM) of ABA. The linear plots of (**B**) the SERS intensity versus the ABA concentration (from 0.04 µM to 40 µM) in the logarithmic range and (**D**) absorbance ratio (A620/A528) versus the ABA concentration (from 0.4 µM to 80 µM) in the logarithmic range. Error bars show the standard deviation of three repeated experiments.

Under the optimized conditions, the constructed dual-signal Apt sensor was used for the quantitative detection of ABA. Figure 5A showed that the SERS intensity of signal molecules gradually increased with the increase in ABA concentration logarithm. Figure 5B indicates that there was a good linear relationship between ABA concentration (from 0.04 µM to 40 µM) logarithm and SERS intensity. The modified curve y = 4082 + 1492Logx was determined, R^2^ was 0.9998, and the calculated LOD was 17.6 nM. Figure 5C,D showed that the A620/A528 increased with the increase in ABA concentration in the range of 0.04 µM to 80 µM, indicating that ABA aggregation intensity increased. Figure 5D showed that the A620/A528 showed a good linear relationship with the logarithm of ABA concentration (from 0.4 µM to 80 µM). The linear equation was y = 0.0884Logx + 0.4841, R^2^ was 0.9963, and the calculated LOD was 36 nM. Meanwhile, in order to facilitate the comparison, this method was compared with traditional methods reported in the past in terms of characteristics, and the results were shown in Table 1. Although the detection limit of the SERS/LSPR sensor was slightly higher than that of the previous method, this method did not require a chemical bond modification process, and the operation was simple and time-saving. Moreover, the output of the two signals verified each other, making the detection result more reliable.

### 3.5. The Selectivity of the Sensor

**Figure 6:**
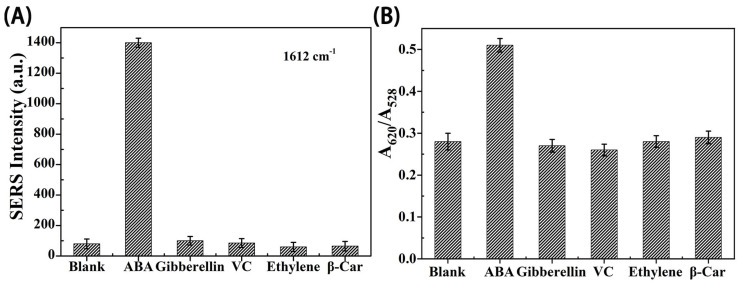
The SERS intensity (**A**) and A620/A528 (**B**) of dual-signal output aptamer sensor for blank, ABA, GA3, VC, Ethylene, and β-Car. The concentration of ABA was 2 µM, while other interfering molecules were 10 µM. Error bars show the standard deviation of three repeated experiments.

In the process of hormone extraction, some interference substances would be produced, such as vitamin C (VC), beta-carotene (β-Car), gibberellin, ethylene, and other hormones. The experimental results in Figure 6A,B showed that, under the condition of ABA concentration of 20µM, while other interfering substances had a concentration of 2 mM, the intensity of SERS and A620/A528 increased with the increase in time. In ABA, vitamin C, β-carotene, gibberellin, and ethylene, only ABA could induce AuNPs aggregation and generate a strong SERS signal, and the absorbance ratio A620/A528 was significantly different from the blank value. This indicates that the sensor has good selectivity.

### 3.6. Abscisic Acid Determination in Real Plant Tissue

To further evaluate the application ability of the biosensor in a variety of plant substrates, ABA was extracted from corn seedlings, cucumber leaves, tomato leaves, tomato fruits, and other plant tissues. The dual-signal aptamer sensor was used to detect ABA, and the detection results were compared with an ELISA kit. The samples used in the experiment came from the laboratory of the College of Agriculture, Henan Agricultural University. The detection results are shown in Table 2. The SERS detection results showed that the maximum error of the ABA content was 13.46%, and the LSPR assay showed that the maximum error of ABA content was 14.01%. These results showed that the results of the two methods were consistent, and the results could be verified by each other. It can be inferred from the experimental results that the aptamer biosensor has great potential in the detection of ABA, ethylene, salicylic acid, and other plant hormones.

## 4. Conclusions

In conclusion, a simple and highly sensitive plant hormone ABA biosensor based on LSPR and SERS was prepared through the specific interaction between Apt and ABA, the aggregation of dispersed AuNPs, and the enhanced plasma coupling with formed SERS. According to the absorbance ratio of two characteristic wavelengths A620/A528 in the absorption spectrum of the detection system and the SERS intensity of the characteristic peak of CV molecule at 1612 cm^−1^, the quantitative evaluation was carried out. Under optimized experimental conditions, in the range of 0.04 µM~80 µM, absorption spectrum analysis showed that the R^2^ of the absorption spectrum was 0.9922, and the LOD was 15 nM. The SERS spectrum had an R^2^ of 0.9922, and the LOD was 13 nM. The linear range and the LOD of the two evaluation methods were almost consistent. In addition, the biosensor has good selectivity for other plant hormones as well as VC and beta-carotene in plant tissues. It also has the ability to detect the real ABA extracted from the leaves of common crops, compared with the ELISA method. The maximum relative error was 13.46% in SERS and 14.01% in LSPR. The proposed SERS/LSPR dual-signal biosensor has three advantages. (1) There are two kinds of signals results there were output, SERS and LSPR, which were mutually verified and more reliable. (2) It eliminated the need for chemical modification, and the detection system was simpler. (3) The Apt sequence in the sensor can be changed to detect other substances, which can be considered as a general detection method.

## Figures and Tables

**Table 1:** Comparison of the detection results of the LSPR/SERS dual-signal aptamer sensor with some existing methods for abscisic acid detection.

Number	Method	Detection Range	LOD	References
1	HPLC	4.43–14.2 nM	4.43 nM	[28]
2	Electrochemical immunoassay	10–5000 ng/mL	1 ng/mL	[29]
3	ELISA	0.01–10 pM	0.01 pM	[30]
4	Fluorescence	1 µM–1 mM	<1 µM	[31]
5	LSPR	1 nM–10 μM	0.51 nM	[32]
6	SERS	1 fM–10 nM	0.67 fM	[13]
7	SERS + LSPR	SERS: 0.1 pM–0.1 nM LSPR: 1 pM–10 nM	13 nM 15 nM	

**Table 2:** Comparison of experimental results of ABA determination in real plant samples by dual-signal SERS/LSPR aptamer sensor and ELISA (n = 3).

Number	Sample	ELISA (µM)	SERS (µM)	(Relative Error, RE) (%)	LSPR (µM)	RE (%)
1	Cucumber leaf	1.66	1.76	6.02	1.77	6.57
2	Genetically modified (GM) cucumber leaf	1.76	1.87	6.25	1.88	7.01
3	Tomato seed	1.67	1.45	13.2	1.90	**14.01**
4	Tomato leaf	1.43	1.57	9.79	1.55	8.23
5	GM tomato seed	2.39	2.23	6.69	2.57	7.34
6	GM tomato leaf	1.56	1.35	**13.46**	1.77	13.76
7	Tobacco	1.41	1.56	10.64	1.56	10.98
8	GM tobacco	2.13	2.36	10.79	2.38	11.67
9	Wheat leaf	2.05	1.89	7.80	2.21	7.89
10	Wheat seed	5.24	4.98	4.96	5.52	5.32
11	Corn leaf	2.09	2.21	5.74	2.21	5.93
12	Corn seed	2.23	2.12	4.93	2.35	5.34

## Data Availability

The data generated or derived during this research process have been included in the main manuscript or Supplementary Information.

## Supplementary Materials

The following supporting information can be downloaded at: https://www.mdpi.com/article/10.3390/bios16030152/s1.
