# Supplementary material for: A SERS/LSPR Dual-Signal Aptamer Sensor for Abscisic Acid Detection Based on Unmodified Gold Nanoparticles
Source: Biosensors (Basel). 2026 Mar 10;16(3):152. doi: 10.3390/bios16030152 (PMC13024280; doi:10.3390/bios16030152)
Supplement: Supplementary file 1 [file biosensors-16-00152-s001.zip › biosensors-4120557-supplementary.pdf]

Supplementary Materials

A SERS/LSPR Dual-Signal Aptamer Sensor for Abscisic Acid Detection Based on Unmodified Gold Nanoparticles

Yanyan Zhang [1,2], Junjuan Shang [1,*], Linze Li [1,2], Mengying Du [1,2], Hao Zhang [1,2,*] and Jiandong Hu [1,2,3,*]

[1] College of Mechanical and Electrical Engineering, Henan Agricultural University, Zhengzhou 450002, China; zyanyan0923@henau.edu.cn (Y.Z.); lilinze@henau.edu.cn (L.L.); dmy@stu.henau.edu.cn (M.D.)

[2] Henan International Joint Laboratory of Laser Technology in Agricultural Sciences, Zhengzhou 450002, China

[3] State Key Laboratory of Wheat and Maize Crop Science, Zhengzhou 45002, China

* Correspondence: shangjunjuan@henau.edu.cn (J.S.); hao.zhang@henau.edu.cn (H.Z.); jdhu@henau.edu.cn (J.H.)

1.1 The method for synthesizing AuNPs

AuNPs with an average diameter of about 37 nm were prepared by the reduction of $HAuCl_4 \cdot 4H_2O$ using trisodium citrate. In brief, 500 μL of 1% $HAuCl_4 \cdot 4H_2O$ was added to 49.5 mL ultrapure water in a 100 mL conical flask under slight magnetic stirring. The solution was heated to boiling and kept for 2 min at 120°C. Then, 1 mL of 1% $C_6H_5Na_3O_7 \cdot 2H_2O$ was added quickly under vigorous magnetic stirring for 5 minutes. The colorless solution gradually turned gray, blue, black, and, finally, gradually stabilized to purple. The obtained solution was centrifuged at 12,000 rpm for 10 minutes, and then the sediment was dispersed in the same volume of ultrapure water.

Four kinds of gold seeds with different diameters were synthesized. The morphology of AuNPs can be controlled by changing the addition level of trisodium citrate by adding different doses of 1% trisodium citrate (0.8mL, 1mL, 1.2mL, and 1.5mL) to 50 mL boiled 0.01% $HAuCl_4 \cdot 4H_2O$. As the dosage of trisodium citrate 1% increased from 0.8mL to 1mL, 1.2mL and 1.5mL, AuNPs showed spherical morphology and the average diameter decreased from about 55 nm to 37nm, 28 nm and 18 nm, respectively.

1.2 Methods for obtaining extracts from plant substrates

The fresh samples of 0.2g cucumber, tomato, wheat and other common crops were ground into homogenate with 2mL extracting solution (containing 80% methanol and 1mmol/L butylated hydroxytoluene) and transferred into a 10mL centrifuge tube. Then, the mortar was rinsed twice with 3mL extracting solution, which was then transferred into the centrifuge tube. After shaking, the samples were put into a refrigerator at 4°C for 6-8h and centrifuged at 4000 rpm for 10 minutes to remove the supernatant. The sediment was dried with N_2 flushing, the methanol in the extract was removed, and then the sample was diluted to 10 mL. The content of wheat hormone ABA was determined by an ELISA kit and an SERS/LSPR dual-signal aptamer sensor.
